# Whole-Genome Sequencing and *De Novo* Assembly of 61 Staphylococcus pseudintermedius Isolates from Healthy Dogs and Dogs with Pyoderma

**DOI:** 10.1128/MRA.00152-21

**Published:** 2021-04-22

**Authors:** O. Francino, D. Pérez, J. Viñes, R. Fonticoba, S. Madroñero, G. Meroni, P. Martino, S. Martínez, A. Cusco, N. Fàbregas, L. Migura-García, L. Ferrer

**Affiliations:** a SVGM, Molecular Genetics Veterinary Service, Universitat Autònoma de Barcelona, Bellaterra, Barcelona, Spain; b Department of Animal Medicine and Surgery, Universitat Autònoma de Barcelona, Bellaterra, Barcelona, Spain; c Vetgenomics, Edifici EUREKA, PRUAB, Bellaterra, Barcelona, Spain; d Department of Biomedical, Surgical and Dental Sciences–One Health Unit, Milan, Italy; e Hospital Escuela de Pequeños Animales (HEPA), Facultad de Ciencias Veterinarias de la Universidad Nacional del Centro de la Provincia de Buenos Aires, Tandil, Buenos Aires, Argentina; f IRTA, Centre de Recerca en Sanitat Animal (CReSA, IRTA-UAB), Bellaterra, Barcelona, Spain; g Animal Medicine and Surgery, Universitat Autònoma de Barcelona, Bellaterra, Barcelona, Spain; Indiana University, Bloomington

## Abstract

We have *de novo* assembled and polished 61 Staphylococcus pseudintermedius genome sequences with Nanopore-only long reads. Completeness was 99.25%. The average genome size was 2.70 Mbp, comprising 2,506 coding sequences, 19 complete rRNAs, 56 to 59 tRNAs, and 4 noncoding RNAs (ncRNAs), as well as CRISPR arrays.

## ANNOUNCEMENT

Staphylococcus pseudintermedius is a common microorganism of canine skin (1, 2) and the leading cause of pyoderma in dogs (3, 4). In this study, we aimed to retrieve S. pseudintermedius high-quality genome sequences from healthy dogs and dogs with pyoderma using a *de novo* assembly and polishing strategy with Nanopore-only long reads.

Samples were obtained by rubbing a sterile swab on the skin of healthy dogs (*n* = 22; H) and the nonlesional skin of a dog with pyoderma (*n* = 6; DH) or from the pustules of dogs with pyoderma (*n* = 33; D). After culture in blood agar at 37°C for 24 hours, colonies were seeded in 3 ml of brain heart infusion (BHI) broth at 37°C for 16 hours. DNA was extracted with a ZymoBIOMICS DNA miniprep kit (Zymo Research, Irvine, CA, USA). DNA quality and quantity were determined using a Nanodrop 2000 spectrophotometer and Qubit double-stranded DNA (dsDNA) broad-range (BR) assay kit (Fisher Scientific SL, Madrid, Spain). The sequencing libraries were prepared with the rapid barcoding sequencing kit (SQK-RBK004; Oxford Nanopore Technologies, UK). Twelve barcoded samples were loaded into a MinION FLO-MIN106 v9.4.1 flow cell (Oxford Nanopore Technologies Ltd.) and sequenced into a MinION Mk1B instrument. The fast5 files were basecalled and demultiplexed and adapters trimmed with Guppy 4.0.11 (Oxford Nanopore Technologies) (--config dna_r9.4.1_450bps_hac.cfg) (--config configuration.cfg --barcode_kits SQK-RBK004 --trim_barcodes; min_score threshold default 60). Reads with a quality score lower than 7 were discarded. Run summary statistics were obtained with Nanoplot 1.27 (5) (--N50 --fastq).

Samples assigned to S. pseudintermedius by WIMP (6) were *de novo* assembled using Flye 2.7.1 (7) (--nano-raw --genome-size 2.6m --plasmids --trestle). After minimap 2.17 alignment (8), the resulting contigs were polished with Racon 1.4.13 (https://github.com/lbcb-sci/racon) and Medaka 1.0.3 (https://nanoporetech.github.io/medaka/) (medaka_consensus; -m r941_min_high_g360). Genome completeness was assessed with CheckM 1.1.1 (lineage_wf) (9). Circlator 1.5.5 was used to identify the origin (10) (fixstart --min_id 70). Genomes were annotated with NCBI Prokaryotic Genome Annotation Pipeline (PGAP) (11). Multilocus sequence types (MLSTs) were assigned with software MLST 2.0. and database 2.0.0 (https://cge.cbs.dtu.dk/services/MLST-2.0/) (12).

Nanopore sequencing allowed successful *de novo* assembly and polishing of 61 S. pseudintermedius isolates (Table 1). The average read *N*_50_ value was 4,223.38 bp for 2,848,339.50 reads per flow cell (237,361.63 per barcoded sample). The mean genome coverage was 235× (39× to 664×), with an average contig *N*_50_ value of 2.6 Mbp (1.6 Mbp to 2.9 Mbp). The average genome completeness was 99.25% (98.6% to 99.4%, except for HSP082), which is close to previous results with hybrid assemblies (13). The number of contigs per isolate ranged from 1 to 12 (median, 2). The main contig was circular for 47 isolates. The average genome size of S. pseudintermedius was 2.70 Mbp (2.51 to 2.99 Mb), comprising 2,506 coding sequences (CDS; 2,271 to 2,901), 19 complete rRNAs (6 to 7 5S, 6 16S, and 6 23S rRNA genes), 56 to 59 tRNAs, and 4 noncoding RNAs (ncRNAs), as well as CRISPR arrays (0.5; range from 0 to 2). Pangenome analyses of isolates from healthy and diseased individuals will help unravel the differences, if any, that exist between commensal and pathogenic S. pseudintermedius populations.

**TABLE 1:** Characteristics and accession numbers for high-quality genome assemblies from 61 Staphylococcus pseudintermedius isolates^*a*^

Isolate ID^*b*^	MLST^*c*^	Source^*d*^	Yr of isolation	Country of isolation	Assembly ID (accession version no.)	Assembly level^*e*^	Genome accession no.	Genome assembly	No. of contigs	*N*_50_ (contigs) (bp)	Genome size (all contigs) (bp)	No. of CDS (total)	COV^*f*^ (×)	Comp^*f*^ (%)	Cont^*f*^ (%)
DG040	Unknown	Cf, A, SP	2017	Italy	GCF_016482145.1	Contig	JAENDF000000000	Circular	3	2,527,299	2,564,892	2,369	664	99.43	0.00
DG050	Unknown	Cf, A, SP	2016	Italy	GCF_016482445.1	Contig	JAENDE000000000	Circular	4	2,594,061	2,637,713	2,429	326	99.43	0.57
DG059	Unknown	Cf, A, SP	2017	Italy	GCF_016481825.1	Contig	JAENDD000000000	Circular	2	2,568,059	2,573,568	2,364	223	99.29	0.00
DG062	ST71	Cf, A, SP	2016	Italy	GCF_016482085.1	Contig	JAENDC000000000	Linear	10	2,096,046	2,991,046	2,901	253	99.43	2.84
DG063	ST71	Cf, A, SP	2016	Italy	GCF_016482045.1	Contig	JAENDB000000000	Linear	10	2,099,753	2,981,523	2,891	256	99.43	0.57
DG064	ST71	Cf, A, SP	2017	Italy	GCF_016455205.1	Compl	CP066718	Circular	1	2,895,060	2,895,060	2,764	361	99.43	0.57
DG066	ST71	Cf, A, SP	2017	Italy	GCF_016482585.1	Contig	JAENDA000000000	Circular	3	2,804,246	2,808,032	2,639	213	99.43	0.57
DG067	ST71	Cf, A, SP	2017	Italy	GCF_016482535.1	Contig	JAENCZ000000000	Linear	3	2,890,387	2,896,399	2,776	533	99.43	0.57
DG071	ST71	Cf, A, SP	2012	Italy	GCF_016482425.1	Contig	JAENCY000000000	Linear	3	2,800,617	2,807,986	2,644	85	99.43	0.57
DG072	ST71	Cf, A, SP	2012	Italy	GCF_016455165.1	Compl	CP066717	Circular	1	2,837,133	2,837,133	2,690	304	99.43	0.57
DG076	ST258	Cf, A, SP	2012	Italy	GCF_016482525.1	Contig	JAENCX000000000	Circular	5	2,732,273	2,780,144	2,573	323	99.43	0.85
DG077	ST71	Cf, A, SP	2016	Italy	GCF_016481985.1	Contig	JAENCW000000000	Circular	4	2,789,419	2,791,496	2,624	479	99.43	0.57
DG078	ST71	Cf, A, SP	2012	Italy	GCF_016481935.1	Contig	JAENCV000000000	Circular	3	2,792,180	2,799,396	2,637	208	99.43	0.57
DG081	ST301	Cf, A, SP	2012	Italy	GCF_016482005.1	Contig	JAENCU000000000	Circular	3	2,676,449	2,694,747	2,519	353	99.43	0.00
DG082	ST258	Cf, A, SP	2017	Italy	GCF_016481945.1	Contig	JAENCT000000000	Circular	2	2,623,528	2,626,557	2,396	259	99.43	0.00
DG089	ST71	Cf, A, SP	2012	Italy	GCF_016481925.1	Contig	JAENCS000000000	Circular	2	2,793,862	2,797,937	2,628	281	99.43	0.57
DG093	ST258	Cf, A, SP	2017	Italy	GCF_016481905.1	Contig	JAENCR000000000	Circular	2	2,640,063	2,648,891	2,433	39	99.43	0.00
DG094	ST71	Cf, A, SP	2017	Italy	GCF_016481855.1	Contig	JAENCQ000000000	Circular	2	2,840,672	2,849,103	2,675	191	99.43	0.57
DG099	Unknown	Cf, A, SP	2017	Italy	GCF_016455185.1	Chrom	CP066716	Linear	1	2,623,014	2,623,014	2,416	168	99.43	0.57
DSP020	ST71	Cf, A, SP	2013	Spain	GCF_016455145.1	Compl	CP066715	Circular	1	2,793,830	2,793,830	2,637	213	99.15	0.57
DSP021	ST71	Cf, A, SP	2013	Spain	GCF_016455225.1	Compl	CP066714	Circular	1	2,795,724	2,795,724	2,637	197	99.43	0.57
DSP022	Unknown	Cf, A, SP	2014	Spain	GCF_016481865.1	Contig	JAENCP000000000	Circular	3	2,718,542	2,767,901	2,555	174	98.86	0.28
DSP024	ST611	Cf, A, SP	2014	Spain	GCF_016482205.1	Contig	JAENCO000000000	Circular	2	2,758,929	2,762,026	2,595	289	99.43	0.57
DSP025	ST71	Cf, A, SP	2014	Spain	GCF_016482155.1	Contig	JAENCN000000000	Circular	6	2,801,923	2,805,515	2,641	295	99.43	0.57
DSP026	ST503	Cf, A, SP	2014	Spain	GCF_016455085.1	Compl	CP066713	Circular	1	2,567,628	2,567,628	2,387	303	99.43	0.00
DSP027	Unknown	Cf, A, SP	2014	Spain	GCA_016455285.1	Compl	CP066712	Circular	1	2,717,194	2,717,194	2,537	352	99.43	0.28
DSP028	Unknown	Cf, A, SP	2019	Spain	GCF_016482125.1	Contig	JAENCM000000000	Circular	2	2,569,147	2,575,420	2,367	238	98.86	0.00
DSP029	ST258	Cf, A, SP	2019	Spain	GCF_016481265.1	Contig	JAENBQ000000000	Linear	8	2,413,604	2,723,805	2,517	270	99.43	0.00
DSP030	Unknown	Cf, A, SP	2018	Argentina	GCF_016455265.1	Compl	CP066711	Circular	1	2,612,059	2,612,059	2,386	164	99.43	0.00
DSP032	ST1631	Cf, A, SP	2019	Argentina	GCA_016482035.1	Contig	JAENCL000000000	Linear	3	1,369,210	2,670,199	2,449	119	98.66	0.57
DSP034	ST1827	Cf, A, SP	2018	Argentina	GCF_016455025.1	Compl	CP066710	Circular	1	2,550,368	2,550,368	2,319	204	99.43	0.00
DSP035	Unknown	Cf, A, SP	2019	Argentina	GCF_016481845.1	Contig	JAENCK000000000	Linear	2	2,640,488	2,642,865	2,489	117	99.43	0.00
DSP036	ST71	Cf, A, SP	2019	Spain	GCF_016482105.1	Contig	JAENCJ000000000	Circular	7	2,849,760	2,927,015	2,755	129	99.43	0.57
DHSP041	ST71	Cf, A, H	2019	Spain	GCF_016482265.1	Contig	JAENBW000000000	Linear	3	2,849,717	2,909,751	2,748	123	99.41	0.57
DHSP042	ST71	Cf, A, H	2019	Spain	GCF_016482345.1	Contig	JAENBV000000000	Linear	6	2,822,064	2,930,017	2,768	72	99.43	0.57
DHSP043	ST71	Cf, A, H	2019	Spain	GCF_016482305.1	Contig	JAENBU000000000	Linear	12	1,649,358	2,931,289	2,776	66	99.43	0.57
DHSP044	ST71	Cf, A, H	2019	Spain	GCF_016482225.1	Contig	JAENBT000000000	Circular	4	2,849,602	2,907,542	2,748	132	99.43	0.57
DHSP045	ST71	Cf, A, H	2019	Spain	GCF_016482025.1	Contig	JAENBS000000000	Circular	5	2,849,736	2,915,461	2,742	127	99.41	0.57
DHSP046	ST496	Cf, P, H	2019	Spain	GCF_016481295.1	Contig	JAENBR000000000	Circular	2	2,695,721	2,710,903	2,475	100	99.43	0.00
HSP079	Unknown	Cf, P, H	2019	Spain	GCF_016598895.1	Compl	CP066885	Circular	1	2,585,691	2,587,693	2,337	301	99.43	0.00
HSP080	Unknown	Cf, P, H	2019	Spain	GCF_016598915.1	Compl	CP066884	Circular	1	2,585,570	2,587,506	2,336	199	99.41	0.00
HSP081	Unknown	Cf, P, H	2019	Spain	GCA_016482135.1	Contig	JAENCI000000000	Circular	4	2,617,897	2,621,254	2,467	94	98.58	1.14
HSP082	Unknown	Cf, P, H	2019	Spain	GCA_016481725.1	Contig	JAENCH000000000	Circular	2	2,617,857	2,620,663	2,466	149	96.73	1.14
HSP093	Unknown	Cf, A, H	2019	Spain	GCF_016481735.1	Contig	JAENCG000000000	Circular	3	2,571,836	2,590,335	2,361	274	99.43	0.57
HSP094	Unknown	Cf, A, H	2019	Spain	GCF_016481805.1	Contig	JAENCF000000000	Circular	2	2,567,494	2,570,595	2,337	550	99.43	0.57
HSP095	Unknown	Cf, A, H	2019	Spain	GCF_016482325.1	Contig	JAENCE000000000	Circular	2	2,570,328	2,575,879	2,360	341	99.43	0.57
HSP096	Unknown	Cf, A, H	2019	Spain	GCF_016482245.1	Contig	JAENCD000000000	Circular	3	2,570,206	2,578,330	2,359	89	99.43	0.57
HSP097	Unknown	Cf, A, H	2019	Spain	GCF_016481665.1	Contig	JAENCC000000000	Circular	3	2,570,223	2,575,223	2,356	136	99.43	0.57
HSP118	Unknown	Cf, A, H	2019	Spain	GCF_016455005.1	Compl	CP066709	Circular	1	2,512,855	2,512,855	2,277	313	99.43	0.00
HSP125	ST1248	Cf, A, H	2019	Spain	GCF_016455245.1	Compl	CP066708	Circular	1	2,551,473	2,551,473	2,330	327	98.86	0.00
HSP127	ST1061	Cf, P, H	2019	Spain	GCF_016481685.1	Contig	JAENCB000000000	Circular	2	2,660,509	2,690,618	2,507	162	98.86	1.14
HSP132	Unknown	Cf, P, H	2019	Spain	GCA_016455125.1	Chrom	CP066707	Linear	1	2,515,164	2,515,164	2,275	132	98.72	0.00
HSP134	Unknown	Cf, P, H	2019	Spain	GCA_016455105.1	Compl	CP066706	Circular	1	2,514,594	2,514,594	2,274	146	98.86	0.00
HSP135	Unknown	Cf, A, H	2019	Spain	GCF_016455065.1	Compl	CP066705	Circular	1	2,512,727	2,512,727	2,277	154	98.86	0.00
HSP136	Unknown	Cf, A, H	2019	Spain	GCA_016455045.1	Chrom	CP066704	Linear	1	2,512,726	2,512,726	2,274	104	98.86	0.00
HSP137	Unknown	Cf, A, H	2019	Spain	GCF_016454985.1	Compl	CP066703	Circular	1	2,512,757	2,512,757	2,271	224	99.43	0.00
HSP138	Unknown	Cf, A, H	2019	Spain	GCF_016454965.1	Compl	CP066702	Circular	1	2,512,830	2,512,830	2,277	227	98.86	0.00
HSP140	Unknown	Cf, P, H	2019	Spain	GCF_016482365.1	Contig	JAENCA000000000	Circular	2	2,594,004	2,597,272	2,387	296	99.24	0.00
HSP141	ST257	Cf, P, H	2019	Spain	GCF_016482355.1	Contig	JAENBZ000000000	Circular	4	2,615,633	2,622,529	2,450	150	99.43	0.57
HSP142	Unknown	Cf, P, H	2019	Spain	GCF_016482405.1	Contig	JAENBY000000000	Circular	2	2,594,059	2,597,098	2,393	461	99.43	0.00
HSP143	Unknown	Cf, P, H	2019	Spain	GCF_016482235.1	Contig	JAENBX000000000	Linear	6	2,537,079	2,779,670	2,589	293	98.86	0.57

a Obtained from the lesional skin of 33 dogs with pyoderma (33 D), the nonlesional skin of a dog with pyoderma (6 DH), and 6 healthy dogs (22 H).

b ID, identifier.

c MLST, multilocus sequence type; ST, sequence type.

d Cf, Canis lupus familiaris; A, abdominal skin swab; P, perioral skin swab; SP, superficial pyoderma; H, healthy.

e Compl, complete genome; Chrom, chromosome.

f COV, coverage; Comp, completeness; Cont, contamination.

### Data availability.

The standardized strain descriptions and accession numbers are presented in Table 1; the genome sequence assemblies and genomic data are publicly available in DDBJ/ENA/GenBank under BioProject no. PRJNA685966 with the accession numbers CP066702 to CP066718, CP066884, CP066885, and JAENBQ000000000 to JAENDF000000000. The versions described in this paper are the first versions. The raw data are available from the Sequence Read Archive (SRA) under the same BioProject no., PRJNA685966.
